# Comparison of a novel automated DiaSys procalcitonin immunoassay with four different BRAHMS-partnered immunoassays

**DOI:** 10.1016/j.plabm.2022.e00274

**Published:** 2022-04-12

**Authors:** Abass Eidizadeh, Mechthild Wiederhold, Moritz Schnelle, Lutz Binder

**Affiliations:** Institute for Clinical Chemistry/Interdisciplinary UMG Laboratory, University Medical Center Goettingen, Robert-Koch-Str. 40, 37075, Goettingen, Germany

**Keywords:** Sepsis, Procalcitonin, PCT, Immunoassay, PETIA, Calibration, Architect, Liaison, Alinity, Cobas, Roche, Abbott, DiaSys, Diasorin, Standardization, Reference material, Comparison, Bacterial infection

## Abstract

**Objectives:**

Procalcitonin (PCT) is an important biomarker of sepsis and respiratory infections. Various automated immunoassays for measuring PCT in patient plasma are available in medical laboratories. However, due to a lack of international reference material for PCT, the assays are not always comparable.

**Design and methods:**

In this study, we compared a new turbidimetric immunoassay from DiaSys, measured on the Abbott Architect c16000 and Alinity c, with four BRAHMS-associated chemiluminescence immunoassays (Abbott Architect i2000SR, Alinity i, Roche Cobas e411 and DiaSorin Liaison XL) using 120 random patient plasma samples from the clinical laboratory routine at the University Medical Center Goettingen.

**Results:**

The DiaSys assay showed clear differences as compared to the BRAHMS-associated assays when measured on Architect c: i.e. 58% positive mean bias vs. Architect i, 67% vs. Cobas and 23% vs. Liaison. As a result, additional 19% our patients would have a suspected bacterial infection, when using PCT values from the DiaSys assay and commonly accepted decision limits. A crosscheck of the DiaSys calibrator on the BRAHMS-associated systems showed a low recovery of the calibrator material (approx. 50%).

**Conclusions:**

Overall, this study shows significant differences between the DiaSys and BRAHMS-associated assays. This could be attributed to a potential DiaSys calibrator problem. This highlights the need for an international reference material for harmonization of the PCT assays.

## 1 Introduction

Procalcitonin (PCT), a 116 amino acid precursor peptide of the hormone calcitonin, is a valid diagnostic biomarker in sepsis and respiratory infections with different applications [1,2]. An increase in PCT expression in parenchymal tissue can typically be observed in severe bacterial, but not or significantly less, respectively, in viral infections; thus, measurement of plasma PCT concentrations is an important diagnostic tool for clinicians to distinguish between infections from a bacterial versus viral origin [3,4]. In this context, high PCT plasma concentrations were shown to be associated with higher mortality in critically ill patients [5], and recently PCT assessment in plasma from COVID-19 patients was demonstrated to provide prognostic information [6,7]. In addition, plasma PCT measurements are frequently used for antibiotic therapy monitoring which leads to a more efficient use of antibiotic drugs; this, in turn, has beneficial economic effects and reduces the generation of multi-resistant pathogens [8,9].

The physiological plasma concentration of PCT is very low at <0.05 μg/L. In case of a respiratory tract infection, different suggested PCT concentration cutoffs exist to determine the probability of a bacterial origin: PCT >0.25 μg/L: possible, PCT >0.5 μg/L: probable and PCT >1 μg/L: likely [2,10,11]. Sepsis can be considered relatively likely, along with clinical symptoms, once plasma PCT concentrations exceed 2 μg/L, and severe sepsis when values are >10 μg/L [12]. PCT values below 0.5 μg/L could suggest that antibiotic therapy may not be necessary [4]. For a correct interpretation of PCT measurements, it is obvious that the clinical presentation of the respective patients have to be considered.

The clinical relevance of PCT as biomarker requires accurate analytical methods, which should also be comparable between different laboratories. However, the commercially available assays for automated PCT measurements show marked discrepancies. This is predominantly due to a lack of standardization [[13], [14], [15], [16], [17], [18]]. Some authors consider the BRAHMS-PCT assay a kind of reference method, which all other assays need to be compared to Refs. [14,19]. In this context, the previously mentioned PCT cutoffs for clinical decisions are based on measurements from this particular assay. Indeed, most manufacturers, e.g. Abbott, Roche, Diasorin or Siemens use BRAHMS-associated assays for their automated analyzers; however, further automatable immunoassays, for instance by Diazyme, are also available [13,14]. Recently, DiaSys launched a particle-enhanced turbidimetric immunoassay (PETIA). Such a test could reduce both (i) time to result due to the often faster detection technique by immunoturbidimetry (compared to chemiluminescence) and (ii) costs, compared to the relatively high prices of the BRAHMS PCT assays due to licensing issues [20]. Comparability between the DiaSys assay and the BRAHMS PCT assay on the Kryptor could be demonstrated, indicating good analytical quality of this new assay measured on the Roche Cobas analyzer [20].

The present study was designed to assess analytical comparability between the new DiaSys assay (used on Abbott Architect c and Alinity c) and four BRAHMS-associated assays on different automated analyzers, i.e. Abbott Architect i, Abbott Alinity i, Roche Cobas e411 and DiaSorin Liaison XL.

## 2 Material and methods

### 2.1 Study design

Lithium heparinate plasma (S-Monovette Li-Heparin, Sarstedt AG & Co, Nümbrecht, Germany) samples from 120 patients were randomly selected from clinical laboratory routine at the University Medical Center Goettingen (UMG). Samples were centrifuged for 10 min at 3000 g at 20 °C. Parallel PCT measurements of all samples were carried out using five different automated assays on a total of six analyzers: 1. ARCHITECT BRAHMS PCT on the Architect i2000SR (Abbott Diagnostics, Illinois, USA), a two-step chemiluminescence microparticle immunoassay (CMIA) using acridinium-labeled conjugates. 2. ALINITY i BRAHMS PCT on the Alinity i, also a CMIA (Abbott Diagnostics). 3. DIASYS PCT FS (DiaSys Diagnostic Systems, Holzheim, Germany), a particle-enhanced turbidimetric immunoassay (PETIA) on the Architect c16000 and Alinity c (Abbott Diagnostics). 4. Elecsys® BRAHMS PCT on the Cobas e411 (Roche Diagnostics, Mannheim, Germany), an electro-chemiluminescence immunoassay (ECLIA) in association with a ruthenium complex-conjugated antibody. 5. LIAISON® BRAHMS PCT® II GEN on the Liaison® XL (DiaSorin, Saluggia, Italy), a sandwich chemiluminescence immunoassay using an isoluminol antibody conjugate. The BRAHMS PCT assays all have similar sensitivity. According to the manufacturer, their LoD is 0.02 μg/L (Architect BRAHMS PCT, Elecsys BRAHMS PCT, Liaison BRAHMS PCT). The DiaSys assay has a lower sensitivity with a LoD of 0.2 μg/L (DiaSys PCT). The samples were measured directly on all analyzers in parallel within approx. 2 hours and were not stored, frozen or chilled in between. PCT values obtained in this study covered a wide concentration range from 0.01 to 16.36 μg/L. All assays were used according to the manufacturers' instructions.

### 2.2 Evaluation of imprecision

For each assay, triplicates of two different assay-related PCT quality controls were measured daily for five consecutive days, followed by within-run and total imprecision calculations according to the CLSI EP15-A3 protocol [21]. Results are presented as mean values and coefficients of variation (CV) respectively.

### 2.3 Calibrator crosschecks

Calibrator crosschecks were performed measuring all six levels from the DiaSys assay calibrator (lot number: 2857) in duplicate on the other four analyzers using BRAHMS-associated assays. Mean values are given. Measurements were then compared with the manufacturers' assigned values. Discrepancies between the different assays were intended to indicate potential calibration differences.

### 2.4 Ethical approval

The study was carried out using fresh patient plasma leftover material after completion of routine analysis. All patient samples were used in an anonymized way without consideration of further patient related data. The study was conducted according to the World Medical Association Declaration of Helsinki and is reviewed by the UMG ethics committee (protocol number 19/3/21).

### 2.5 Statistical analysis

Comparison of different PCT assays on different analyzers was undertaken using Passing-Bablok regressions, Pearson's correlation coefficients and Bland-Altman difference plots. Statistical analysis, calculations and graphical presentations were carried out using Microsoft Excel and Microsoft PowerPoint (Microsoft, Redmond, USA).

## 3 Results

### 3.1 Imprecision

The imprecision was assessed using two control levels on each analyzer with the respective PCT assay (Table 1). All assays showed acceptable CVs between 1.8 and 6.4% in total imprecision and between 0.59 and 3.9% in within-run imprecision. The highest imprecision was found on the Liaison_BRAHMS_. The DiaSys assay showed imprecisions between 1.8 and 4.5%. The trueness with respect to the manufacturer's target concentrations was between −7.09 and 6.04%, with the DiaSys assay displaying the highest deviations on both control levels (−5.88% and −7.09% for Alinity c_DIASYS,_ 3.59% and 6.04% for Architect c_DIASYS_).

**Table 1:** Evaluation of imprecision. Results of imprecision analysis on two quality control concentration levels with the DiaSys PCT assay (Architect c, Alinity c) and four BRAHMS-associated PCT assays (Architect i, Alinity i, Cobas, Liaison). Imprecision analysis was performed according to the CLSI protocol. Coefficient of variations (CVs) and trueness to target concentrations are shown (%).

Control-Level	%	Architect iBRAHMS	Architect cDIASYS	Alinity iBRAHMS	Alinity cDIASYS	CobasBRAHMS	LiaisonBRAHMS
1	Within-Run imprecision (CV) (n = 15)	2.6	3.9	3.2	1.5	1.6	2.4
	Total imprecision (CV) (n = 15)	2.6	4.5	2.9	1.8	3.1	6.4
	Trueness (Target in μg/L) (n = 15)	−2.00 (0.20)	3.59 (0.85)	0.33 (0.20)	−5.88 (0.85)	2.94 (0.54)	−0.48 (1.52)
2	Within-Run imprecision (CV) (n = 15)	3.6	2.2	1.8	1.0	1.8	0.59
	Total imprecision (CV) (n = 15)	3.8	2.5	2.4	1.8	1.9	3.9
	Trueness (Target in μg/L) (n = 15)	−6.77 (70.00)	6.04 (12.70)	2.65 (70.00)	−7.09 (12.70)	−2.52 (9.42)	−3.09 (41.00)

### 3.2 Comparison

To investigate the comparability of the DiaSys assay with the common BRAHMS-associated assays, 120 plasma samples were measured immediately after patient routine diagnostics had been completed: four analyzers with BRAHMS-PCT assays (Architect i_BRAHMS_, Alinity i_BRAHMS_, Liaison_BRAHMS_, Cobas_BRAHMS_), and two analyzers with the DiaSys PCT assay (Architect c_DIASYS_ and Alinity c_DIASYS_). The concentration range of the samples was between 0.01 and 16.36 μg/L.

**Fig. 1:**
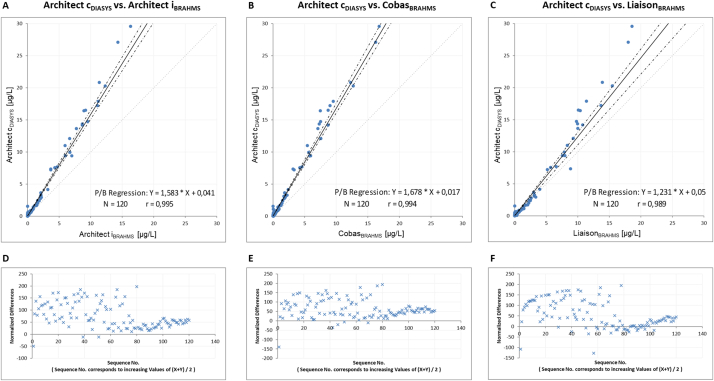
Passing-Bablok regression analysis (A–C) and Bland-Altman difference plots (D–F) on Architect, Cobas and Liaison. Passing-Bablok regression and Bland-Altman difference plots were performed from PCT measurements in 120 plasma samples between DiaSys PCT assay on Architect c and three BRAHMS-associated assays on Architect i, Liaison and Cobas. 95% confidence intervals (CI) are presented as dotted lines. Linear equations with Pearson's correlation coefficients (*r*) are presented in the respective figures. In difference plots, each normalized difference is plotted against the respective sample rank determined by the average of both respective measurements.

**Fig. 2:**
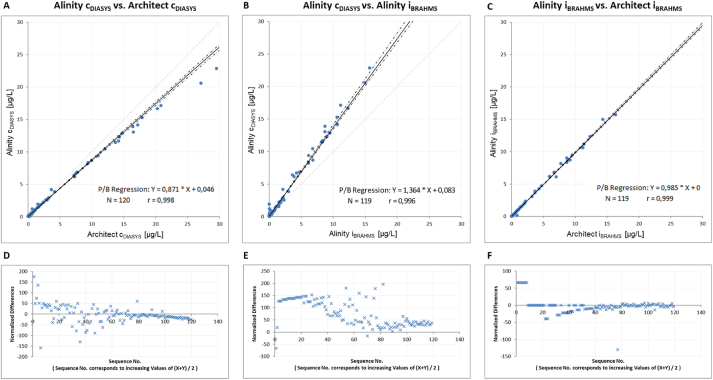
Passing-Bablok regression analysis and Bland-Altman difference plots on Alinity i, Alinity c and Architect. Passing-Bablok regression and Bland-Altman difference plots were performed from PCT measurements in 120 plasma samples between DiaSys PCT assay on Architect c and Alinity c and two Abbott BRAHMS-associated assays on Architect i and Alinity i. 95% confidence intervals (CI) are presented as dotted lines. Linear equations with Pearson's correlation coefficients (*r*) are presented in the respective figures. In difference plots, each normalized difference is plotted against the respective sample rank determined by the average of both respective measurements.

The regression analysis shows a clear positive deviation between the DiaSys assay on the Architect c compared to all BRAHMS-partnered assays (Fig. 1A–F): 58% to Architect i_BRAHMS_, 67% to Cobas_BRAHMS_ and 23% to Liaison_BRAHMS_. The DiaSys assay generally results in higher concentrations compared to all BRAHMS assays (Figs. 1 and figure 2B). Nevertheless, the discrepancy (up to 36%) between the BRAHMS and DiaSys assays was also apparent on the Alinitys (Fig. 2B). On the other hand, the DiaSys assays (Alinity c_DIASYS_ vs. Architect c_DIASYS_) and the BRAHMS assays (Alinity i_BRAHMS_ vs. Architect i_BRAHMS_) showed good comparability among themselves between the Architect and Alinity analyzers (figure 2A and C). However, the correlation coefficient was found to be consistent for all comparisons (Pearson's *r* between 0.989 and 0.999).

Furthermore, we wanted to assess how comparable the assays are at low PCT concentrations in the range of the clinical decision limits (supplement Fig. 1, Fig. 2). In concentrations up to 2 μg/L, the differences between the BRAHMS assays and the DiaSys PCT assay were still noticeable (supplement figure 1A-F), but less pronounced (overestimated up to 33% to Architect i_BRAHMS_, 40% to Cobas_BRAHMS_ and less than 1% to Liaison_BRAHMS_), also between the Alinitys (up to 25%; supplement figure 2B). Among themselves, the DiaSys assays (Alinity c_DIASYS_ vs. Architect c_DIASYS_) and the BRAHMS assays (Alinity i_BRAHMS_ vs. Architect i_BRAHMS_) showed good comparability in concentrations up to 2 μg/L (supplement figure 2A,D and C,F). Although the correlation was found to be much less consistent (Architect i_BRAHMS_ vs. Architect c_DIASYS_: *r* = 0.913; Cobas_BRAHMS_ vs. Architect c_DIASYS_: *r* = 0.883; Liaison_BRAHMS_ vs. Architect c_DIASYS_: *r* = 0.881).

If > 0.5 μg/L was used as a clinical decision limit, 19% more patients would have suspected a bacterial infection when measuring the PCT on Architect c_DIASYS_ (n = 51) compared to Architect i_BRAHMS_ (n = 43). The same applies when compared to the Cobas_BRAHMS_ (n = 43). For the Liaison_BRAHMS_ (n = 46) it would have been only 7%.

Considering the clinical decision limit of 2 μg/L to determine a possible sepsis, a comparison of Architect i_BRAHMS_ (n = 27) to Architect c_DIASYS_ (n = 29) would still lead to 11% overdiagnosed patients and possibly unnecessary application of antibiotic therapy, when using clinical decision limits derived from BRAHMS assay (for more details see Table 2).

**Table 2:** Comparison of positive findings using different clinical decision limits between BRAHMS PCT assays and DiaSys PCT assay. Shown are the number of values higher than or equal to the clinical cutoff of 2.00 μg/L or 0.50 μg/L for suspected sepsis and bacterial infection when using the various BRAHMS-associated assays (Architect i, Alinity i, Cobas, Liaison) in comparison to DiaSys PCT assay (on Architect c and Alinity c) of all 120 patient samples.

Cutoff	BRAHMS PCT Assay				DiaSys PCT Assay	
	Architect i	Alinity i	Cobas	Liaison	Architect c	Alinity c
≥2.00 μg/L	27	27	23	31	29	29
≥0.50 μg/L	43	42	43	46	51	49

### 3.3 Calibrator crosscheck

Comparative analysis showed that PCT concentration measurements using the DiaSys PCT assay were generally higher as compared to the BRAHMS-associated assays. We suspected a systematic error affecting the calibration, and therefore performed calibrator crosschecks. Six levels of the DiaSys PCT calibrator were measured on four analyzers with BRAHMS-associated assays and the recovery from the manufacturer's target concentration was evaluated (Table 3). Throughout all concentrations, the recovery was low (e.g. in low concentration levels up to 4.24 μg/L: only 46% on Cobas_BRAHMS_ and 55% on Architect i_BRAHMS_; in higher levels (55 μg/L): 44% on Architect i_BRAHMS_ and 48% on Cobas_BRAHMS_). Only the Liaison_BRAHMS_ showed at least a recovery of 69% at high concentrations. This is also reflected in a better regression between the Liaison_BRAHMS_ and Architect c using the DiaSys assay. These findings point towards a calibration bias for the DiaSys assay.

**Table 3:** Calibrator crosscheck. Calibrator crosscheck was performed, measuring the six level calibrator from DiaSys PCT assay with the BRAHMS-associated assays (Architect i, Alinity i, Cobas, Liaison).

Calibrator	Level	Target (μg/L)	Architect i (μg/L)	Alinity i (μg/L)	Cobas (μg/L)	Liaison (μg/L)
DiaSys PCT	1	**0.00**	<0.02	<0.02	0.04	<0.02
	2	**0.84**	0.61	0.63	0.54	1.17
	3	**4.24**	2.35	2.50	1.95	4.25
	4	**12.90**	5.89	6.21	5.07	9.87
	5	**22.60**	9.64	10.14	8.93	16.70
	6	**55.00**	24.68	25.84	26.59	38.40

## 4 Discussion

Sepsis and bacterial infections are a global health system challenge. In 2017, 11 million sepsis-related deaths of hospitalized patients were registered worldwide, that is 19.7% of all global deaths, with a mortality of 52.8% [22]. PCT can be helpful in diagnosing sepsis as well as monitoring response to antibiotic treatment [10]. Reliable and fast PCT detection in the medical laboratory is therefore essential. With respect to the use of PCT decision limits, diagnostic quality and inter-laboratory comparability depend on the analytical performance of the assays used.

In the present study we wanted to analyze the comparability of the new DiaSys PCT PETIA with the commonly used BRAHMS-associated assays (detection based on chemiluminescence) on four analyzer systems (Architect i, Alinity i, Cobas and Liaison). To the best of our knowledge, this is the first study comparing the new DiaSys assay with BRAHMS-associated assays commonly used in many laboratories.

Up to now, only one study assessed the analytical performance of the DiaSys PCT assay [20]. Dupuy et al. used the DiaSys assay on the Roche Cobas 8000 system and compared it with the BRAHMS PCT assay on the Kryptor. The comparability with the BRAHMS PCT assay on the Kryptor was excellent. Only a slight overestimation of approx. 15% compared to the Kryptor could be demonstrated. Notably, the deviation was found to be even less than 1% in PCT concentrations below 2 μg/L. An overall discordance of 19% at the clinical decision limits was found between the DiaSys assay and the Kryptor; the authors concluded that the DiaSys assay shows good comparability to the Kryptor and is suitable for clinical use.

In our study, the imprecision of the DiaSys assay was acceptable and comparable to those of the BRAHMS assays. The trueness was slightly lower at Architect c_DIASYS_ and Alinity c_DIASYS_ in comparison to the BRAHMS assays, but acceptable from an analytical point of view. These data are in line with the imprecision data from the previous study [20].

When comparing the DiaSys assay with the other assays, clear discrepancies in PCT results could be observed: generally, the DiaSys assay measured higher PCT concentrations, e.g. 58% as compared to Architect i_BRAHMS_, 67% as compared to Cobas_BRAHMS_ and at least 23% as compared to Liaison_BRAHMS_. Despite these discrepancies, correlation coefficients were consistent. In the low concentration range close to the clinical decision limits, the deviation was less pronounced, but the correlations were worse. We were not able to confirm the findings of Dupuy et al. in comparison with other BRAHMS-associated assays, although measured on Abbott analyzers. However, differences to the study by Dupuy et al. must also be mentioned here: the DiaSys assay was measured there with the Roche c502 Cobas 8000 system, while we used Abbott analyzer. Also, no information about calibrator lot are given, so that it cannot be checked here whether Dupuy et al. used the same calibrator lots as we did. Nevertheless, sample handling and number of samples are similar. Although Dupuy et al. show good comparability of the DiaSys assay with the Kryptor BRAHMS PCT assay, all BRAHMS-associated PCT assays themselves show good comparability with Kryptor, although clear differences between them have already been shown several times [23].

We suspected a calibrator bias behind the systematically higher concentrations from the DiaSys assay in the inter-assay comparison. Therefore, we performed a calibrator crosscheck, and we found a low recovery of all calibrator levels from the DiaSys assay on all BRAHMS assays. The manufacturer's information on the DiaSys calibrator indicates that the calibrator concentrations can be traced back to the Roche Cobas e411. However, our measurements on the Cobas showed a recovery of only 64.28%. We could not prove the traceability proclaimed by the manufacturer.

As in our previous PCT study [17], we showed a good regression of the BRAHMS-associated assays on Architect and Cobas, but a clear deviation of Liaison as compared to the other two platforms. This could most likely be attributed to a general calibration bias of the Liaison assay, similar to our observation presented in this study regarding the DiaSys assay.

The results from the present investigation again underline the need for an internationally available PCT reference material. Based on such material, PCT assays from different companies can be traced back to a uniform calibration resulting in comparable PCT results in patient samples. Such uniform calibration will allow the use of clinical decision limits derived from extensive studies, independent of the assay available. Clinical decision-making using PCT assays with inconsistent calibration materials will continue to produce potentially contradictory decisions and may endanger patients. Using the DiaSys assay, 19% of our patients would be overdiagnosed and would have the suspected diagnosis of a bacterial infection, which could lead to unnecessary therapy.

Also the BRAHMS-associated assays are not homogeneous and comparable with one another, as already shown in several studies. Lippi et al. demonstrated a bias between 0.2% up to 38.6% between 10 automated PCT immunoassays, although all BRAHMS-associated assays showed good comparability with the Kryptor [18]. In addition, Dipalo et al. reported good alignment between BRAHMS-associated assays and the Kryptor [13]. Although the common BRAHMS-associated PCT assays are comparable to the Kryptor, discrepancies can be seen between different systems. In this regard, Soh et al. noted the greatest discrepancies between the Architect and the Cobas assay, although both were found to be comparable to the Kryptor [15]. In contrast, other studies reported relevant differences between PCT measurements using Architect or Cobas assays vs. Kryptor [16,24]. These study results are in part contradictory and confusing. The question regarding the origin of these contradictions has not yet been answered. Considering that PCT – different to CRP (C-reactive protein) – is not very stable (unless lyophilized), it may be speculated that during production, storage, transport and handling in the laboratory, calibration material might get compromised to individually varying degrees. This hypothesis may at least partially explain differing results from various investigations.

It should be noted that the discordance of the DiaSys assay, when compared to others, is not so large like other competitive products such as the Diazyme assay, which even differs significantly from the Kryptor assay [14,18,20,24].

The currently used PCT clinical decision limits for decisions regarding antibiotic therapy and monitoring were established with the BRAHMS PCT assay on Kryptor [25]. This must be taken into account with the observed deviations between the BRAHMS-associated assays. The use of these clinical decision limits in non-BRAHMS-associated assays, such as the DiaSys assay, should be critically questioned. This could lead to overdiagnosis or result in elongated and imprecise antibiotic therapy, which in turn may contribute to the development of multi-resistant germs [26].

As a side note, to the best of our knowledge, this work is the first using the Abbott BRAHMS-associated PCT assay on Abbott's new generation of analyzers, the Alinity i. The Alinity i correlated very well with the Architect i, Cobas and Liaison, even at low concentrations in the range of the clinical decision limits. The Passing-Bablok regressions showed very good comparability between Alinity i, Architect i and Cobas, with hardly any deviations throughout all concentration ranges.

Overall, this was the first time that a detailed comparative study between various conventional BRAHMS-associated assays with the new DiaSys PETIA was carried out on clinical routine samples. We could see significant differences, with the DiaSys assay showing systematically higher concentrations than the BRAHMS assay. We suspect a matter in calibration. This must be taken into account when changing to the DiaSys assay, also with regard to the clinical decision-making limits.

The absence of international reference material and the lack of a diagnostic standard in the determination of PCT is a serious reason for the deficiency of comparability of the various PCT immunoassays. A recently founded international committee of the International Federation of Clinical Chemistry and Laboratory Medicine (IFCC) is now trying to harmonize the PCT assays by introducing a reference material and a reference method [27]. The results of this collaboration will hopefully further improve PCT-guided antiinfective treatment.

## Funding

This study received no external funding.

## Author contributions

Conceptualization: A.E. and L.B.; Methodology: M.W. and A.E.; Software: A.E.; Validation: A.E., M.S., M.W. and L.B.; Formal Analysis: A.E. and M.S.; Investigation: M.W.; Resources: A.E. and M.W.; Data Curation: A.E. and L.B.; Writing – Original Draft Preparation: A.E.; Writing – Review & Editing: A.E., M.S. and L.B.; Visualization: A.E.; Supervision: L.B.; Project Administration: A.E. All authors have read and agreed to the published version of the manuscript.

## Declaration of competing interest

The authors have declared that no conflict of interest exists.
