# Supplementary material for: Comparison of a novel automated DiaSys procalcitonin immunoassay with four different BRAHMS-partnered immunoassays
Source: Pract Lab Med. 2022 Apr 12;30:e00274. doi: 10.1016/j.plabm.2022.e00274 (PMC9026940; doi:10.1016/j.plabm.2022.e00274)
Supplement: Multimedia component 1 [file mmc1.docx]

**Supplement Material**

**„Comparison of a novel automated DiaSys procalcitonin immunoassay with four different BRAHMS-partnered immunoassays“**

**Eidizadeh et al.**

**
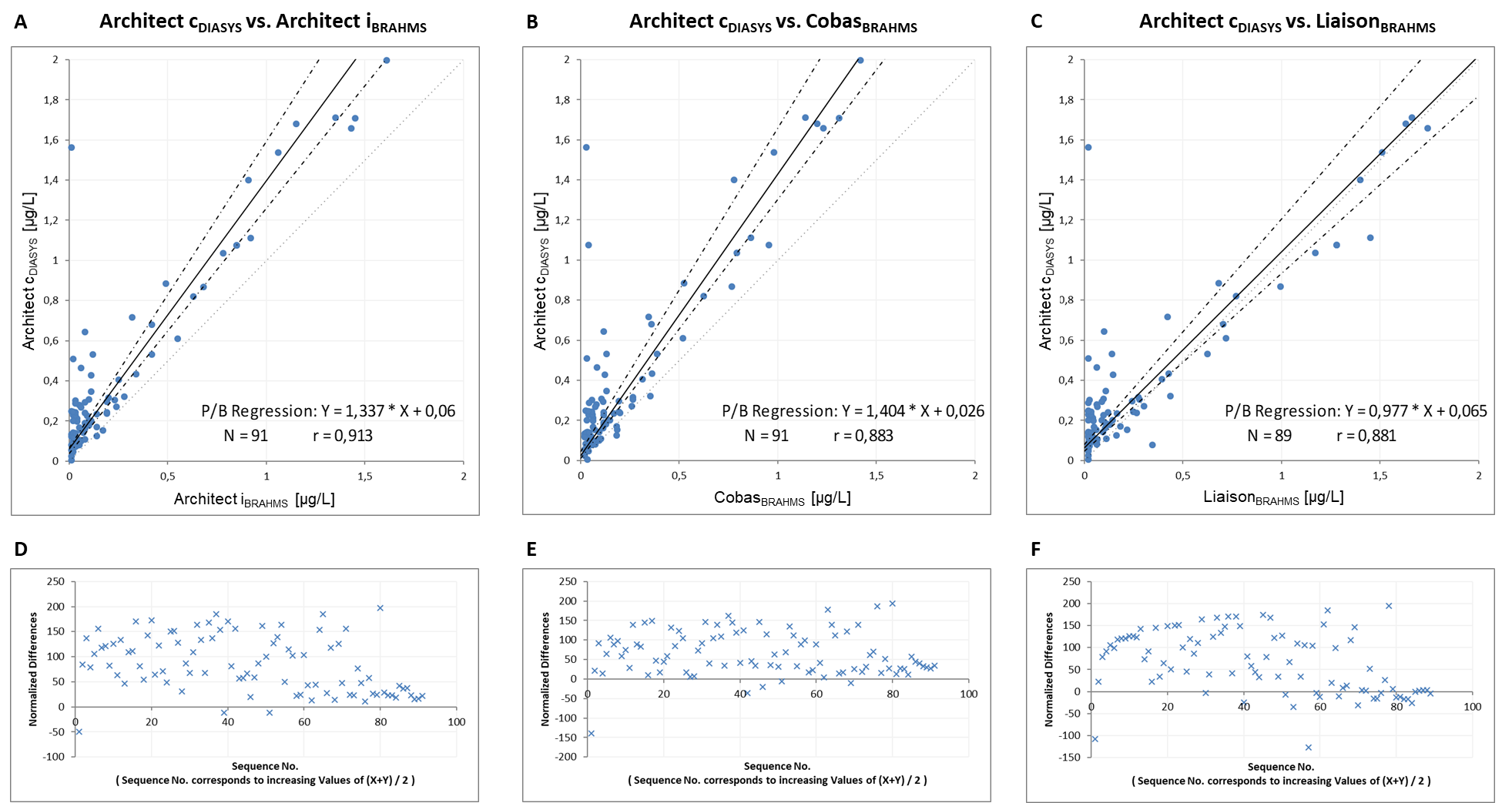
**

***Supplement Figure 1:* Passing-Bablok regression analysis (A-C) and Bland-Altman difference plots (D-F) on Architect, Cobas and Liaison for PCT concentrations ≤ 2 µg/L.** Passing-Bablok regression and Bland-Altman difference plots were performed from PCT measurements in patient plasma samples between DiaSys PCT assay on Architect c and three BRAHMS-associated assays on Architect i, Liaison and Cobas. 95% confidence intervals (CI) are presented as dotted lines. Linear equations with Pearson's correlation coefficients (*r*) are presented in the respective figures. In difference plots, each normalized difference is plotted against the respective sample rank determined by the average of both respective measurements.


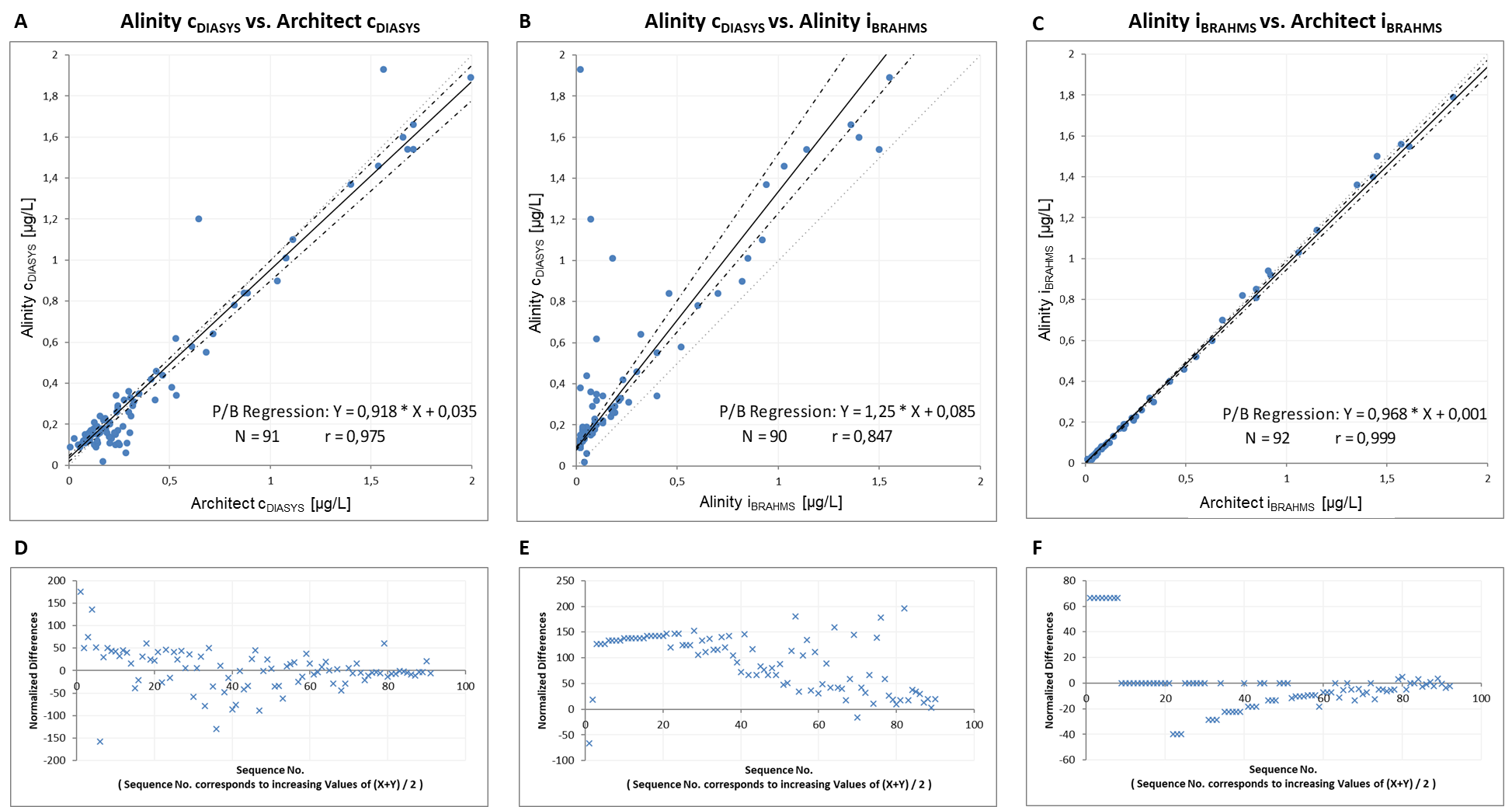


***Supplement Figure 2:*** **Passing-Bablok regression analysis and Bland-Altman difference plots on Alinity i, Alinity c and Architect for PCT concentrations ≤ 2 µg/L.** Passing-Bablok regression and Bland-Altman difference plots were performed from PCT measurements in patient plasma samples between DiaSys PCT assay on Architect c and Alinity c and two Abbott BRAHMS-associated assays on Architect i and Alinity i. 95% confidence intervals (CI) are presented as dotted lines. Linear equations with Pearson`s correlation coefficients (*r*) are presented in the respective figures. In difference plots, each normalized difference is plotted against the respective sample rank determined by the average of both respective measurements.
